# Improved peridynamic model and its application to crack propagation in rocks

**DOI:** 10.1098/rsos.221013

**Published:** 2022-10-19

**Authors:** Luming Zhou, Shu Zhu, Zhende Zhu, Shuang Yu, Xinghua Xie

**Affiliations:** ^1^ Key Laboratory of Ministry of Education for Geomechanics and Embankment Engineering, Hohai University, Nanjing 210098, People's Republic of China; ^2^ Jiangsu Research Center for Geotechnical Engineering Technology, Hohai University, Nanjing 210098, People's Republic of China; ^3^ Jiangsu Health Vocational College, Nanjing, Nanjing 211800, People's Republic of China; ^4^ State Key Laboratory of Hydrology-Water Resources and Hydraulic Engineering, Nanjing Hydraulic Research Institute, Nanjing 210029, People's Republic of China

**Keywords:** peridynamics, improved peridynamics model, rock fracture, crack propagation and coalescence

## Abstract

The conventional bond-based peridynamics (BB-PD) model is suitable for simulating the failure mode of homogeneous elastic-brittle materials. However, the strain hardening and subsequent strain softening characteristics of rocks under loading cannot be reflected. In addition, the fracture mechanisms of rock materials under tension and compression are completely different. To solve these problems, this paper proposes an improved BB-PD model using different fracture criteria in the tensile and compression stages of the bond based on previous improved models, and a critical failure condition obeying the Weibull distribution is introduced to reflect the heterogeneity of the rock. The crack propagation processes of an intact rock specimen, rock specimen with a single pre-existing flaw and rock specimens with two and three pre-existing flaws under compressive loading are simulated using the model, and its feasibility is verified by comparing with the results of previous laboratory tests. Next, the effects of the inclination angle and length on the wing crack propagation length are studied. Finally, the changes in the crack aggregation modes under different rock bridge inclination angles are simulated. Eight types of crack aggregation modes are found, and the conditions under which they may occur are analysed. The improved model proposed can effectively simulate the crack propagation and coalescing processes and has a wide application prospect for rock fracture simulations.

## 1. Introduction

A rock is a type of heterogeneous and discontinuous material containing many flaws such as primary cracks of different sizes and directions which has experienced complex geological tectonic processes. The cracks in rocks may cause tunnel collapse, dam cracking, overturning of buildings and other engineering accidents. Therefore, it is of great significance to study the initiation, propagation and coalescence of the cracks in rocks for the prevention and control of natural disasters.

Several physical tests have been conducted on the propagation processes of the rock specimen with a single pre-existing flaw [1–6] and multiple pre-existing flaws with different lengths and inclination angles [7–11]. However, there remain some difficulties in studying rock fractures: (i) to obtain comprehensive and ideal results, it is necessary to perform multiple groups of parallel experiments, which require considerable time and cost; (ii) the local fracture of rock materials occurs in a short duration, making it difficult to obtain the dynamic fracture characteristic parameters such as the crack propagation length and crack coalescence time clearly and completely.

The common numerical methods based on continuum mechanics include the finite-element method (FEM) [12,13], finite difference method (FDM) [14], boundary element method (BEM) [15], the extended finite-element method (XFEM) [16,17] and so on. However, the governing equations of the FEM and FDM are derived from the continuum mechanics, and the stress field at the crack tip is mathematically singular. In addition, FEM needs to remesh at the crack tip when simulating crack propagation. BEM also has the problem of singularity of boundary variables. The XFEM [16,17] can simulate the crack growth by introducing a local strengthening function. However, XFEM has difficulties in simulating multi-crack propagation with complex morphologies [18]. The mesh-less method (MLM) eliminates the mesh dependency and can be roughly divided into two categories: particle methods based on the Lagrange method, such as smoothed particle hydrodynamics [19], and gridless methods based on the Euler method, such as the element-free Galerkin [20]. Compared with the XFEM, the MLM improves the continuity of the interpolation [21,22]; however, it is difficult to apply the essential boundary conditions because the shape functions do not meet the nature of the Kronecker delta function [23,24]. Other MLMs, such as mesh-less local Petrov–Galerkin [25], partition of unity method [26] and so on, also have their own limitations in terms of long calculation time and efficiency.

Common non-continuous numerical methods include the discontinuous deformation analysis (DDA) [27], numerical manifold method (NMM) [28] and discrete element method (DEM) [29,30]. However, the prediction of crack growth mode by DDA is still limited by the division of grid [31]. In NMM calculation, the crack cannot extend into the interior of the element [28]. Some parameters such as friction coefficient and stiffness ratio without clear physical meaning need to be calibrated in the process of DEM calculation, which may have a great influence on the accuracy of the results [32,33].

Silling *et al*. [34–36] proposed the theory of peridynamics (PD) and the corresponding numerical method to simulate the evolution processes of crack initiation, propagation, bifurcation and coalescence in solid structures under a unified mathematical framework. Different from the classical continuum mechanics involving the use of the displacement component derivative, the PD is a non-local continuum mechanics theory, which discretizes the solid into a series of material points containing all the information pertaining to the physical properties in the spatial domain. Based on an integral equation, it avoids the problem that the spatial derivative does not exist or is not unique. The characteristics of the integral equation allow the damage to initiate in multiple parts of the solid material and propagate along any path, without requiring a specific crack propagation criterion. Therefore, the PD overcomes the shortcomings of crack tip singularity, the need for external fracture criteria, the inability to simulate crack initiation and strong grid dependence. Hence, it is widely used to simulate the discontinuous deformation of various materials such as concrete [37–39], glass [40,41], membrane and fibre [42] and other composites [43–45].

The bond-based peridynamics (BB-PD) model has been proved to be effective in simulating the initiation and propagation processes of macrocracks in rock materials with multiple pre-existing flaws with different lengths and angles under uniaxial tensile [46] and compressive loads [47–49]. In addition, several microelastic–brittle plastic constitutive models were also proposed to consider the post-peak stage of a rock mass [50–52]. However, the above studies often assumed that the properties of rock materials are homogeneous and paid more attention to the changes in the rock fracture mode and stress–strain curve; there is a lack of research on dynamic fracture phenomena such as the rock crack propagation length, crack bifurcation and coalescence.

The PD theory can be divided into BB-PD, ordinary state-based PD and non-ordinary state-based PD. The three theories have been proved to be effective in simulating the crack propagation process of rock [46–54]. Despite the limitation of fixed Poisson's ratio, BB-PD theory has fewer parameters and its constitutive model is easier to understand than the other two theories. It is convenient to improve the form of constitutive force function according to the characteristics of strain hardening and strain softening of rock materials. The BB-PD method adopts the microelastic–brittle model, which states that the force between any two material points is equal but opposite in direction. The points are conceptualized to be connected by springs, and the force is linear. When the bond stretch exceeds a critical value, the force suddenly disappears. However, for rock materials, the stress does not disappear suddenly after reaching the peak strength. Moreover, rock deformation has the characteristics of strain hardening first and then strain softening, which is particularly evident under compressive loading. The conventional microelastic little model cannot reflect these characteristics.

In this study, the conventional microelastic–brittle model is improved on the basis of reviewing the characteristics of previous BB-PD models. A microelastic–plastic constitutive model in line with the mechanical properties of rock materials is proposed, and the Weibull distribution function is introduced to reflect the heterogeneity of rocks. The proposed model is used to simulate the crack propagation process of rock specimens containing one, two and three pre-existing flaws, and the proposed model is validated by comparing with the laboratory test results. On this basis, a series of numerical experiments is conducted to simulate the crack propagation modes of rock specimens containing pre-existing flaws with different numbers and inclination angles under uniaxial compression, to study the crack initiation and propagation characteristics and to reveal the differences between the crack propagation length and crack coalescence mode. The results are expected to enrich the content of the PD theory and promote the application of the PD method for the fracture simulation of rock materials.

## 2. Methodology

### 2.1. Classical bond-based peridynamics theory and numerical methods

**Figure 1.**
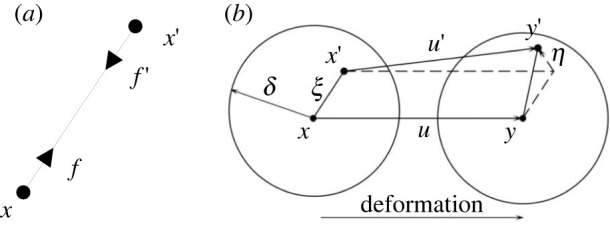
Illustration of variables in a BB-PD model: (*a*) pairwise force function; (*b*) deformation diagram.

As shown in figure 1*a*, the PD theory assumes that at time *t*, particle ***x*** in the object interacts with other particles ***x****’* in a certain area *H_x_* around it through the pairwise force function ***f***. ***f*** is defined as follows:

2.1 $$f=f(u(x^{\prime },t)-u(x,t),x^{\prime }-x),$$

where ***u***(***x***,***t***) and ***u***(***x’***,***t***) are the displacement vectors of material points ***x*** and ***x****’*, respectively.

The motion equation of the material point ***x*** at time *t* is as follows:

2.2 $$\rho \overset{\cdot \cdot }{u}(x,t)=\int_{H_{x}}f(u(x^{\prime },t)-u(x,t),x^{\prime }-x)dV_{x^{\prime }}+b(x,t),$$

where *ρ* is the density of ***x***, ***ü*** is the acceleration vector of ***x***, ***b*** is the applied physical density vector and *dV_x’_* is the infinitesimal volume linked to material point at ***x′***.

The concept of horizon *H_x_* is defined as follows:

2.3 $$H_{x}=H(x,\delta )=\{x\in R:\|x^{\prime }-x\leq \delta \|\},$$

where *δ* is the radius of *H_x_*, which means that the point within the distance *δ* from the material point ***x*** will interact with it.

As shown in figure 1*b*, ***ξ*** and ***η*** are the relative position vector and relative displacement vector of the points ***x*** and ***x****’*.

2.4 $$\begin{array}{rl} \xi = & x^{\prime }-x\\ \eta = & u(x^{\prime },t)-u(x,t) \end{array}\}.$$

The BB-PD model uses stretch ***s*** to represent the bonds deformation between material points, which is defined as follows:

2.5 $$s=\frac{\left\|\xi +\eta \|-\|\xi \right\|}{\left\|\xi \right\|}.$$

The pairwise force function ***f*** is defined as [35] follows:

2.6 $$f(\xi ,\eta )=\left\{ \begin{array}{c} \frac{\xi +\eta }{\left\|\xi +\eta \right\|}c(\xi ,\delta )s\mu (\xi ,x,t),\;\|\xi \|\leq \delta \;\\ 0,\;\|\xi \|>\delta ,\; \end{array}\right.$$

where *c* is the micromodulus function. *μ* is a scalar function, which is the failure criterion of the bond:

2.7 $$\mu (\xi ,x,t)=\left\{ \begin{array}{c} 1\;\mathrm{for}\;s\leq s_{0}\;\\ 0\;\mathrm{for}\;s>s_{0},\; \end{array}\right.$$

where *s_0_* is the critical stretch of the bond. In the microelastic–brittle constitutive model, when the stretch of the bond exceeds *s_0_*, the bond will be broken.

The micromodulus of the two-dimensional plane stress problem can be obtained [55] as follows:

2.8 $$c=\frac{9E}{\pi h\delta^{3}},$$

where *E* is the elastic modulus, and *h* is the thickness.

The critical stretch *s_0_* is determined on the basis of the breaking energy *G*_0_, which means the energy required to break all the bonds per unit fracture area,

2.9 $$G_{0}^{}=2h\int_{0}^{\delta }\int_{z}^{\delta }\int_{0}^{\arccos z/\xi }w_{0}(\xi )\xi d\varphi d\xi dz=\frac{cs_{0}^{2}h\delta^{4}}{4}.$$

Therefore, the critical stretch *s_0_* is

2.10 $$s_{0}^{}=\sqrt{\frac{4G_{0}^{}}{hc\delta^{4}}}.$$

The PD theory has its own failure criteria. The damage is defined by considering the fracture of the bond at a point, and the local damage ***φ*** is defined as follows:

2.11 $$\varphi (x,t)=1-\frac{\int_{H_{x}}\mu (x^{\prime }-x,t)dV_{x^{\prime }}}{\int_{H_{x}}dV_{x^{\prime }}}.$$

**Figure 2.**
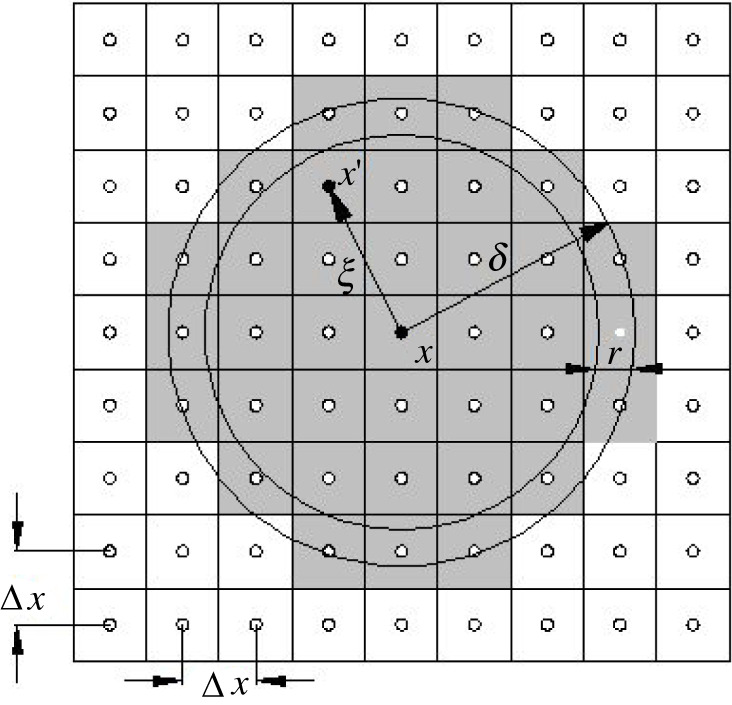
Diagrammatic sketch of discretization.

The collocation method is used to solve equation (2.2). As shown in figure 2, the entire model is evenly discretized into multiple subdomains, and the physical properties of each subdomain are transmitted through the geometric centre point of the subdomain. The distance between any two centre points is Δ*x.* At a certain time *t*, the material point ***x_i_*** interacts with the point ***x_j_*** within the horizon; thus, equation (2.2) can be replaced using Riemann sums, as follows:

2.12 $$\rho \overset{\cdot \cdot }{u_{i}^{t}}=\sum_{j}f(\xi ,\eta^{t})V_{j}+b_{i}^{t},$$

where *V_j_* is the volume of point *x_j_*. The point volume is corrected according to Madenci & Oterkus [56],

2.13 $$V_{j}=\left\{ \begin{array}{c} {\left(\Delta x\right)}^{3},\;\|\xi \|\leq (\delta -r)\;\\ (\frac{\delta +r-\|\xi \|}{2r}){\left(\Delta x\right)}^{3},\;(\;\delta -r)<\|\xi \|\leq \delta \;\\ 0,\;\|\xi \|>\delta ,\; \end{array}\right.$$

where *r* is half the grid spacing Δ*x*.

Equation (2.12) is solved using the explicit central difference scheme

2.14 $$\overset{\cdot \cdot }{u_{i}^{n}}=\frac{u_{i}^{n+1}-2u_{i}^{n}+u_{i}^{n-1}}{\Delta t^{2}},$$

where *n* is the number of time steps. According to Silling *et al*. [35], the value of time step Δ*t* should meet equation (2.15),

2.15 $$\left\{ \begin{array}{c} \Delta t<\sqrt{\frac{2\rho }{\sum_{j}V_{j}|C(\xi )|}}\;\\ C(\xi )=\frac{\partial f}{\partial \eta }(0,\xi )\; \end{array}\right..$$

**Figure 3.**
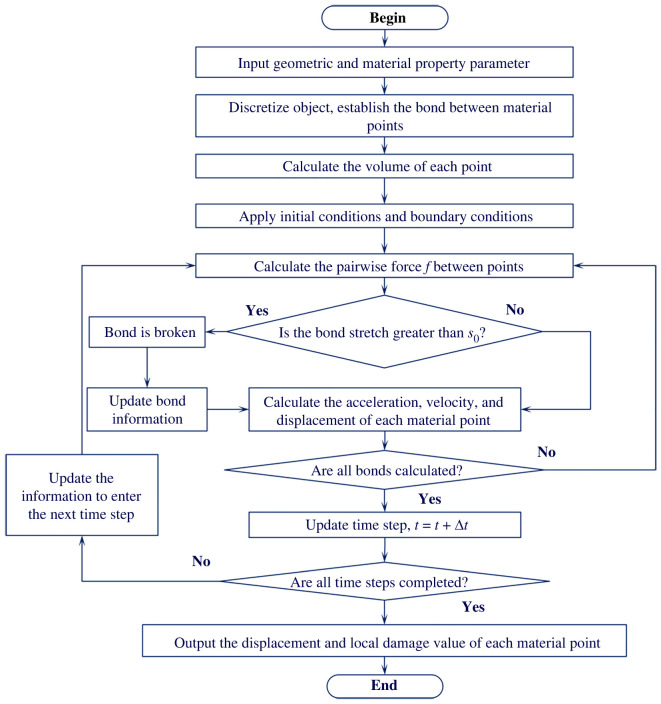
Simulation flowchart of the BB-PD model.

Fortran language is used to realize the above calculation process; figure 3 shows the flowchart of the detailed work.

### 2.2. Existing constitutive model

**Figure 4.**
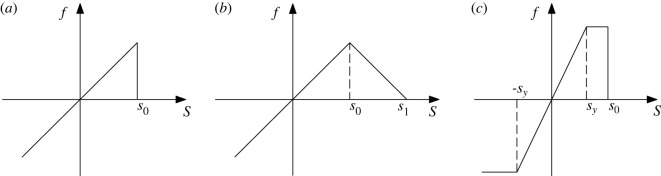
Schematics of existing constitutive models: (*a*) PMB; (*b*) MP; (*c*) EEP.

As shown in figure 4*a*, Silling *et al*. [35] proposed the classic prototype microelastic–brittle model (PMB), which is the basis for improving the other BB-PD models. Gerstle *et al*. [57] proposed a slightly more complex damage model for concrete structures. It is considered that the pairwise force first increases linearly and then decreases linearly with the tensile stretch (as shown in figure 4*b*). This model is called the ‘micropolar peridynamic model (MP)’. Silling *et al*. [58] proposed a Euler form of the ideal elastic–plastic material model (EEP). When the absolute value of the bond stretch exceeds the constant *s*_y_, the pairwise force will remain unchanged whether in the tensile stage or in the compression stage until the tensile stretch exceeds the critical elongation *s_0_* (as shown in figure 4*c*). This model can simulate very large deformations, such as strong shock waves and fluid responses.

The PMB model is suitable for simulating the failure characteristics of elastic-brittle materials. The improvement idea of the subsequent constitutive model is to consider the plastic characteristics of the material by changing the mathematical relationship between the force function and the bond stretch. For example, in figure 4*b,c*, different functions are used to reflect the residual strength after the peak value of the material. However, these models also have their own limitations. For example, in figure 4*b*, the change in the bond's constitutive model is only considered in the tensile stage. When the material is subjected to an external load, the bond between the material points may be extended or shortened, which has no absolute correlation with whether the external load is compressive or tensile. In addition, due to the obvious elastic–plastic characteristics of rock under compression load, the constitutive force function linearly increased (PMP and MP) or the function linearly increased to a constant value (EEP) in the compression stage of the previous BB-PD model cannot well reflect the plastic characteristics of the rock. Therefore, it is necessary to improve the constitutive relation of BB-PD model in the compression stage.

### 2.3. Improved bond-based peridynamics constitutive model

**Figure 5.**
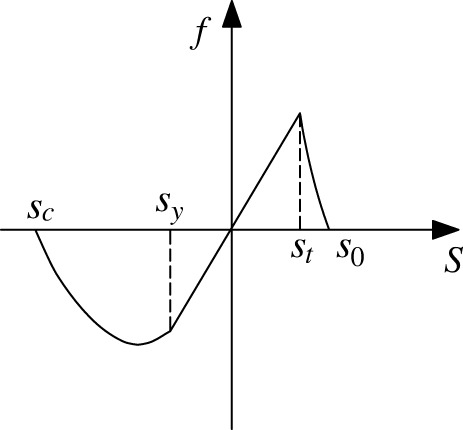
Schematic of the improved BB-PD constitutive model.

Generally, rock materials, whether in compression or tension, show characteristics of strain hardening first and subsequent strain softening. When the stress reaches the maximum value, the material does not fail immediately; in fact, the stress decreases gradually with the increase in the strain until the final failure. The conventional PMB model cannot reflect this feature. Based on the ideas of predecessors and combined with the characteristics of the tensile and compressive curves of rock materials, an improved BB-PD constitutive model is proposed in this paper. The tensile strength of rocks is significantly lower than the compressive strength. The strain hardening stage is not evident under tensile loading, and the stress drops rapidly to a lower level after reaching the tensile strength. Therefore, this study uses a logarithmic function to improve the constitutive model for the bond tensile stage. As shown in figure 5, the pairwise force first increases linearly with the increase in the bond stretch. When the bond stretch exceeds the constant *s*_t_, the pairwise force decreases logarithmically. In the compression stage of the bond, the pairwise force first increases linearly with the increase in the bond stretch. When the absolute value of the bond stretch exceeds the constant *s*_y_, pairwise force is in the form of a quadratic function curve, it corresponds to the characteristics of strain hardening and strain softening of the rock under compression loading.

A damage coefficient *γ* is introduced to modify the pairwise force function. The improved BB-PD constitutive model is expressed as follows:

2.16 $$f(\xi ,\eta )=\gamma (s)\frac{\xi +\eta }{\left\|\xi +\eta \right\|}cs\mu ,$$

and

2.17 $$\gamma (s)=\left\{ \begin{array}{c} 0,\;s\leq s_{c},s\geq s_{0}\;\\ \frac{s-s_{c}}{s_{y}-s_{c}},\;s_{c}<s\leq s_{y}\;\\ 1,\;s_{y}<s\leq s_{t}\;\\ \frac{s_{t}\ln(1+s-s_{0})}{s\ln(1+s_{t}-s_{0})},\;s_{t}<s<s_{0}\; \end{array}\right..$$

where *s*_c_ represents the ultimate compressive value of the bond, which is related to the rock compressive strength *f*_c_, *s*_c_ = −*f*_c_/*E*. *s*_y_ is related to the yield strength *f*_y_, *s*_y_ = −*f*_y_/*E*. *s*_t_ is related to the tensile strength *f_t_*, *s_t_* = *f*_t_/*E*. *s_0_* represents the ultimate tensile stretch of the bond, which is related to the fracture energy *G*_0_. The compressive strength and tensile strength of rock are taken according to the results of laboratory experiments, while the value of fracture energy is determined by reference to the study of Zhu *et al*. [49].

It should be noted that in the improved BB-PD constitutive model, the variation trend of the pairwise force value is controlled by the bond length between material points, but its direction is always parallel to the bond direction, which is the same as the classical BB-PD model. Therefore, the shear force between material points is not considered in the improved BB-PD model. In addition, the expression of each parameter in equation (2.16) can only provide a simple theoretical reference value. When the PD model is used to simulate the rock fracture behaviour, the theoretical value can be modified using the reverse analysis method on the basis of the results obtained from laboratory tests. Finally, the value of the critical stretch within an acceptable range can be obtained [56].

## 3. Model validation

To validate the proposed model, two-dimensional numerical simulations of uniaxial compression tests are conducted on an intact rock specimen, rock specimen with a single pre-existing flaw, and a rock specimen with double and three pre-existing flaws. Their feasibility and applicability are verified by comparing with previous laboratory test results.

Since a rock is a typical heterogeneous material, the crack propagation conditions at each point in the rock may be different. To characterize the heterogeneity of rock materials, the Weibull distribution function is used to realize the randomization of the mechanical properties of the materials,

3.1 $$W(p)=\frac{m}{\;p_{0}}{\left(\frac{\;p}{\;p_{0}}\right)}^{m-1}\exp\left[-{\left(\frac{\;p}{\;p_{0}}\right)}^{m}\right],$$

where *p* represents the distribution parameter value satisfied by each particle (e.g. elastic modulus or critical elongation), *p*_0_ is called the scale parameter, and *m* is called the shape parameter.

**Figure 6.**
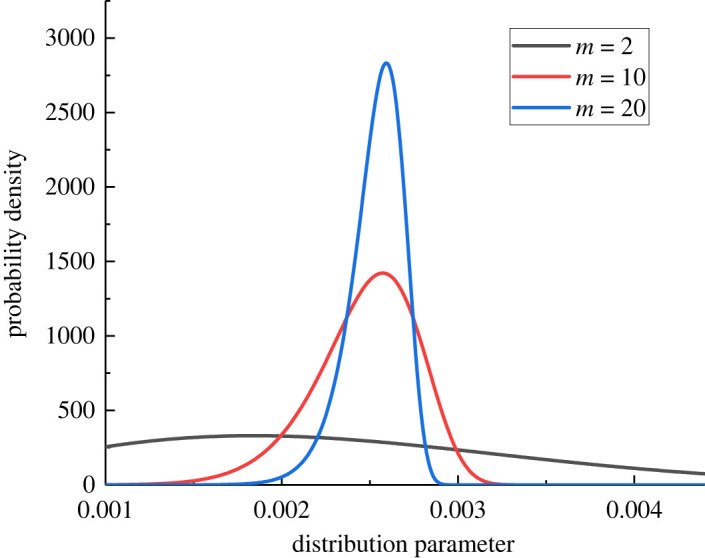
Probability density map of Weibull distribution function.

**Figure 7.**
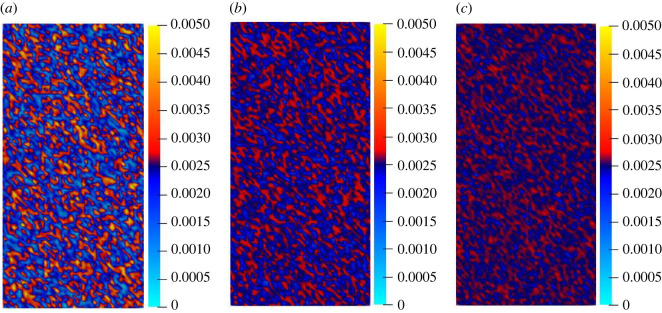
Random distributions of the critical stretch under different homogeneity values: (*a*) *m* = 2; (*b*) *m* = 10; (*c*) *m* = 20.

It is assumed that the critical stretch *s_0_* of each point of rock material obeys the Weibull distribution function that *p*_0_ = 0.0026. Due to the inconsistent size of *s*_0_ of each particle, the mechanical properties of each point will be affected. To ensure that the interaction force between two points is equal, the critical stretch *s*_0_ is taken as the average value of the interacting points, and its fracture judgement criterion is *s* ≥ (*s*_0_(*i*) + *s*_0_(*j*))/2. Figure 6 shows the probability density map of Weibull distribution function when *p*_0_ = 0.0026. Figure 7 shows the random distribution of the critical stretch of rock specimens under different degrees of homogeneity. Combining figures 6 and 7, it can be seen that when scale parameter is a constant, the lower the *m* value, the more discrete the distribution of the critical stretch; the greater the *m* value, the closer the critical stretch is to the mean value.

### 3.1. Compression simulation of intact rock samples

**Figure 8.**
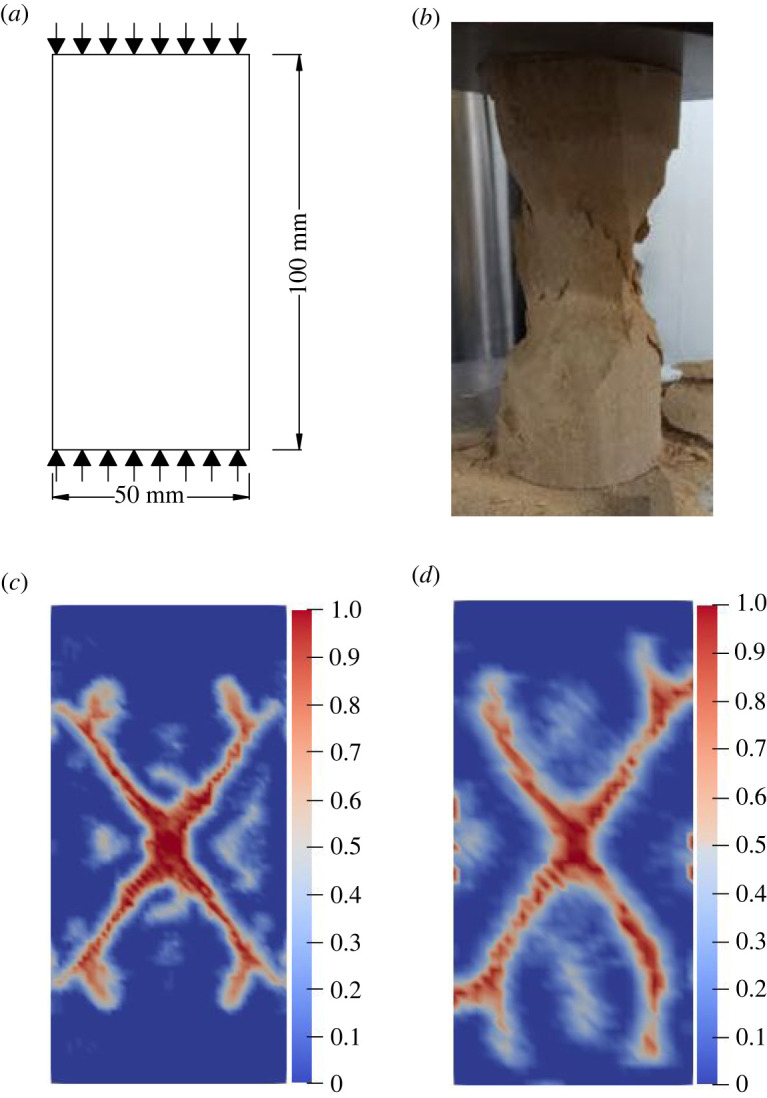
Simulation of a uniaxial compression test for intact rock specimen: (*a*) geometric configuration; (*b*) laboratory test result; (*c*) numerical simulation results of classical model; (*d*) numerical simulation results of improved model.

The experimental model proposed by Gong *et al.* [59] is used to verify that the improved BB-PD constitutive model can effectively simulate the cracking of intact rocks. As shown in figure 8*a*, the dimensions of the two-dimensional model of the complete rock specimen are 50 × 100 mm, and the plane stress method is adopted for the calculation. Table 1 shows the material and PD parameters of the numerical model.

**Table 1.** Material and PD parameters of the numerical model.

parameter	value	parameter	value
length × width (*L* × *D*)	50 × 100 mm	shape parameter (*m*)	40
elastic modulus (*E*)	21 GPa	point spacing (Δ)	0.001 m
Poisson's ratio (*ν*)	1/3	radius of horizon (*δ*)	3.015Δ
density (*ρ*)	2580 kg m^−^^3^	time step (Δ*t*)	5 × 10^−7^ s

Figure 8*b*–*d* shows the results of previous laboratory experiment and numerical simulations. As shown in figure 8*b*, the failure mode is an X-shaped conjugate shear failure, which is one of the most common failure modes of rock materials. Figure 8*c*,*d* is the simulation results of the classical BB-PD model and the improved BB-PD model. The legends in figure 8*c*,*d* show the local damage value, which is calculated from equation (2.11). The closer the value is to 1, the more serious the damage is. In the following figures describing the crack propagation path, the legend has the same meaning as in figure 8*c*,*d*. The X-mode shear crack in the figures is not completely symmetrical, indicating that the Weibull distribution function can better reflect the randomness of rock crack propagation. Through comparison, it is found that when the X-shaped cracks obtained by the classical BB-PD model extends to the left and right ends of the specimen, the position of the cracks reaching the left and right ends is farther from the upper and lower ends of the specimen, while the position of the X-shaped cracks obtained by the improved BB-PD model when it extends to the left and right ends of the specimen is closer to the upper and lower ends of the specimen, which is also more consistent with the results of laboratory experiment. In addition, the symmetry of crack propagation results obtained by the classical BB-PD model is more obvious, because its constitutive force function increases linearly, while the constitutive force function of the improved BB-PD model is a combination of quadratic function, primary function and logarithmic function, so it shows more obvious heterogeneity.

### 3.2. Compression simulation of rock specimen with single pre-existing flaw

**Figure 9.**
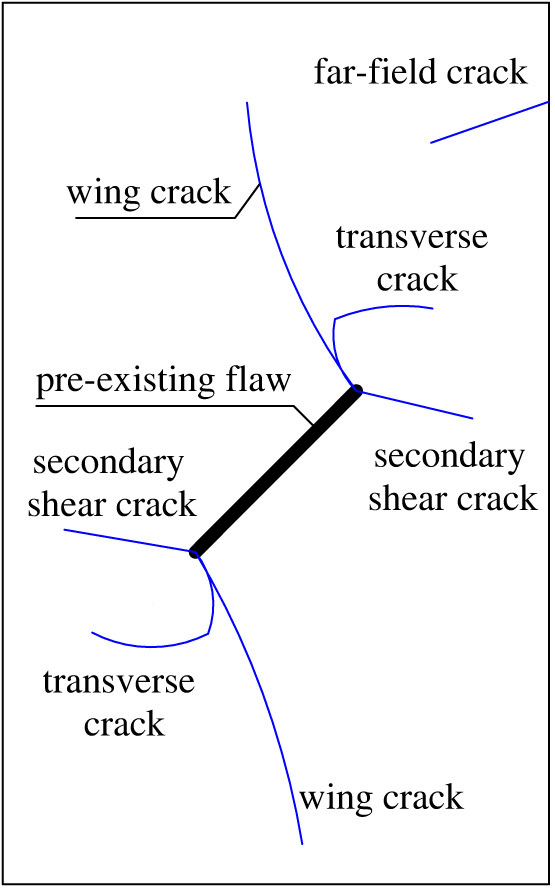
Schematic of crack type.

Figure 9 shows the types of cracks on the rock specimen material. The blue lines represent the various types of cracks. The pre-existing flaw is located at the centre of the rock, and two types of cracks may sprout at both ends of the pre-existing flaw: a wing crack and a secondary shear crack. The wing crack is a tensile crack that arises from the end of the pre-existing flaw and propagates to the maximum compression direction of the rock specimen in a relatively stable manner. Secondary shear cracks are produced by shear and are typically coplanar or quasi-coplanar with the pre-existing flaw. When the wing crack initially propagates for a certain distance, it may turn and expand approximately laterally due to the existence of other pre-existing flaw or cracks. This type of crack is called transverse crack in this paper. In addition, there are far-field cracks far away from the pre-existing flaw generated from the left or right sides of the rock under the action of the shear force.

**Figure 10.**
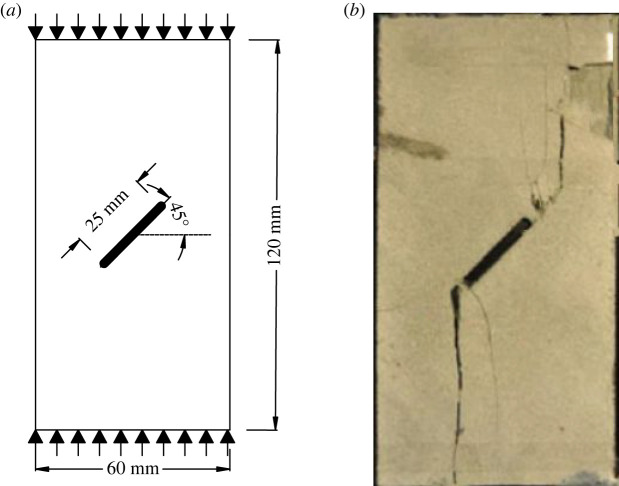
Uniaxial compression test of rock with single flaw: (*a*) geometric configuration; (*b*) laboratory experiment results.

As shown in figure 10*a*, the experimental model proposed by Yang & Jing [60] is used to verify that the improved BB-PD constitutive model can effectively predict the crack generation type and propagation path. The dimensions of the two-dimensional model are 60 × 120 mm, the length of pre-existing flaw is 25 mm and the inclination angle *α* is 45°. The specimen is discretized into 7200 material points. The elastic modulus *E* = 28.56 GPa, mass density *ρ* = 2620 kg m^−^^3^ and shape parameter *m* = 10. The other parameters are the same as those described in §3.1.

**Figure 11.**
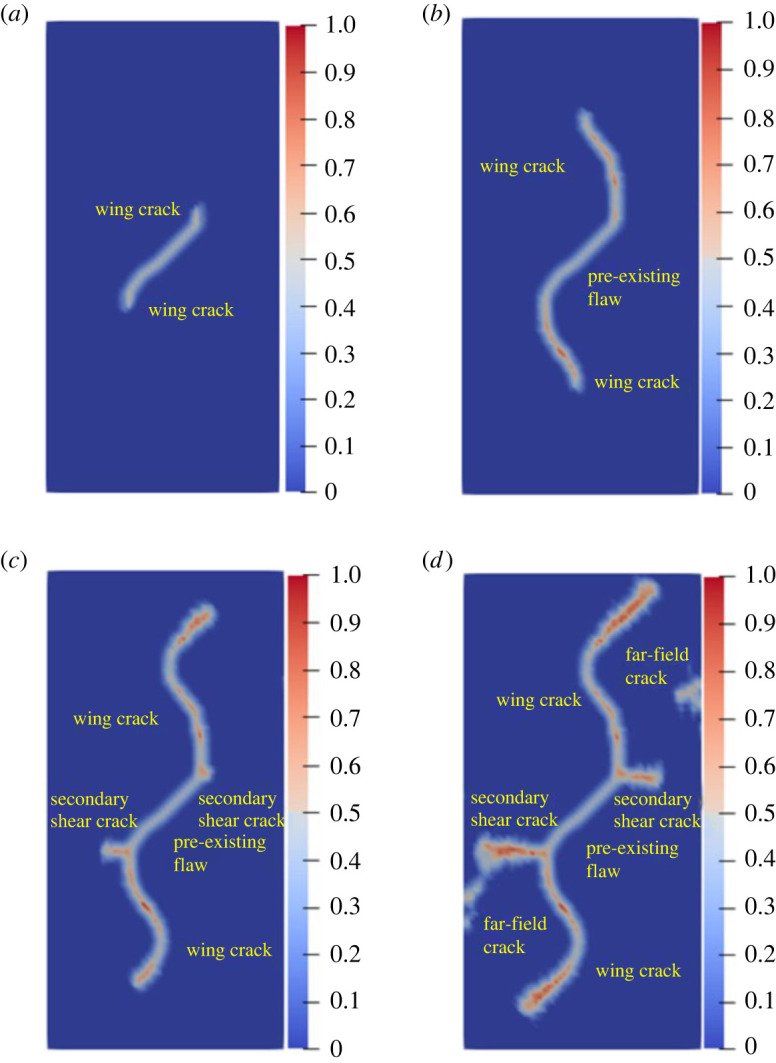
Numerical simulation results of rock with single flaw: (*a*) step 80; (*b*) step 1000; (*c*) step 1610; (*d*) step 1640.

Figure 10*b* shows the previous experimental results. Figure 11 shows the results of numerical simulations. Figure 11*a* shows that the wing cracks first appear at both ends of the pre-existing flaw. The wing cracks appeared at the step 80, which is mainly due to tensile stress and gradually expand to the upper and lower ends of the rock as time progresses. At the step 1630, secondary shear cracks caused by shear appeared at the left and right ends of the pre-existing flaw, mainly exhibiting transverse propagation. The far-field crack appeared at the step 1640. The simulation results are in good agreement with the result of the previous laboratory tests

### 3.3. Compression simulation of rock specimen with double pre-existing flaws

**Figure 12.**
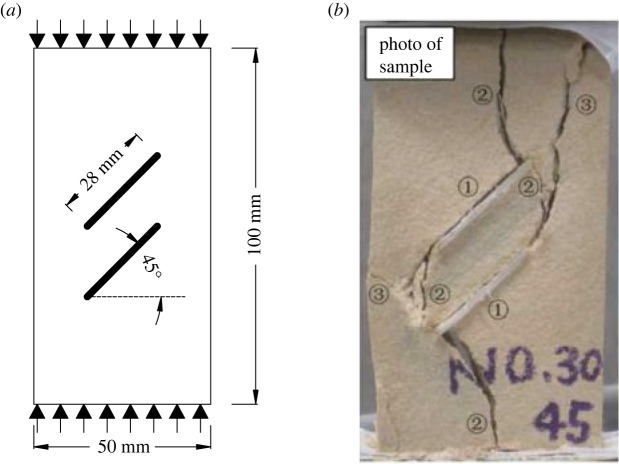
Uniaxial compression test of rock with double flaws: (*a*) geometric configuration; (*b*) laboratory test results.

As shown in figure 12*a*, the experimental model proposed by Yang *et al.* [61] is used to verify that the improved BB-PD constitutive model can effectively predict crack coalescence. The dimensions of the two-dimensional model are 50 × 100 mm, the length of the two pre-existing flaws is 28 mm and the inclination angle is 45°. The specimen is discretized into 5000 material points, rock elastic modulus *E* = 4.71 GPa, mass density *ρ* = kg m^−3^ and shape parameter *m* = 100. The other parameters are the same as those described in §3.1.

**Figure 13.**
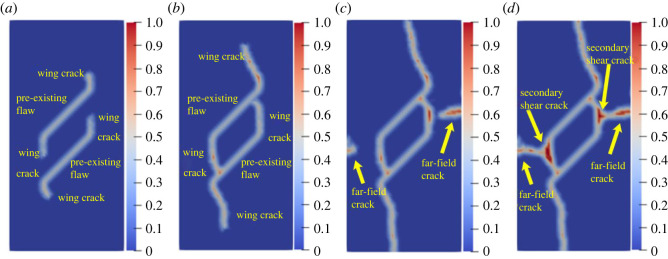
Numerical simulation results of rock with double flaws: (*a*) step 100; (*b*) step 500; (*c*) step 710; (*d*) step 790.

Figure 12*b* shows the previous experimental results. Figure 13 shows the results of numerical simulations. Figure 13*a* shows that in the initial stage of loading, the crack propagation mode of the rock with the double pre-existing flaw is similar to that of the rock with the single pre-existing flaw: independent wing cracks are generated at their respective ends. The wing crack gradually expands to another flaw, and the first crack coalescence occurs at the step 500. After the first coalescence, the wing crack continues to expand to the end of the specimen. At the step 710, secondary shear cracks are initiated at the middle of the coalesced wing crack, and far-field shear cracks are generated at the end of the rock specimen. With the continuous growth of the shear crack, the secondary shear crack is connected with the far-field crack at the step 790, which leads to the second coalescence of the crack and forms a closed shear failure ring. Compared with the experimental result, the numerical simulation can clearly simulate each crack and the damage degree at a certain point in the test, and the crack propagation position is the same as that in the test.

### 3.4. Compression simulation of rock specimen with three pre-existing flaws

**Figure 14.**
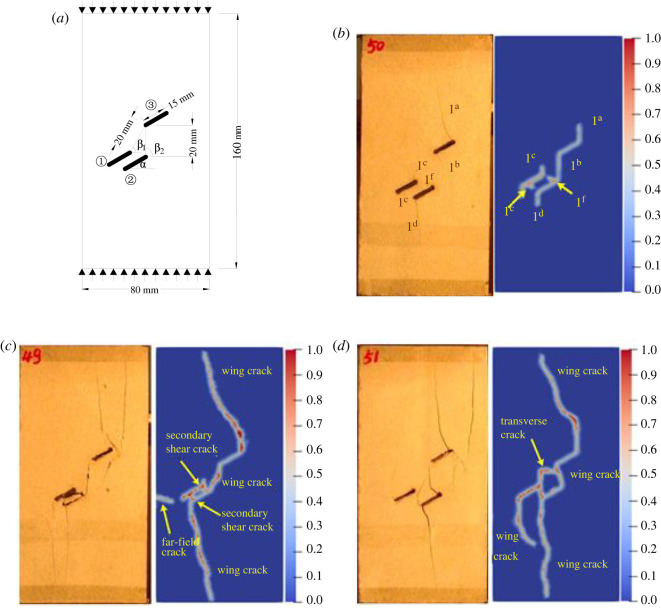
Simulation of uniaxial compression test for rock with three flaws: (*a*) geometric configuration; (*b*) rock bridge inclination *β*_2_ = 90°; (*c*) rock bridge inclination *β*_2_ = 75°; (*d*) rock bridge inclination *β*_2_ = 105°.

To further verify the effectiveness of the improved BB-PD model in simulating the crack propagation process of rock materials with multiple pre-existing flaws, the uniaxial compression test of the rock specimen with three pre-existing flaws with different rock bridge inclination angles is simulated using the experimental model established by Yang [62], as shown in figure 14*a*. The dimensions of the two-dimensional model are 80 × 160 mm. The three pre-existing flaws are numbered as flaw ①, flaw ② and flaw ③ from left to right. The flaw length is 15 mm, the inclination angle *α* is 30°, and the rock bridge inclination *β*_1_ of flaw ① and flaw ③ is fixed at 60°. The rock bridge spacing of flaw ① and flaw ③, flaw ② and flaw ③ is fixed at 20 mm. The specimen is discretized into 12 800 material points, rock elastic modulus *E* = 29 GPa, mass density *ρ* = 2650 kg m^−3^ and shape parameter *m* = 10. The other parameters are the same as those described in §3.1.

The crack propagation of the rock bridge *β*_2_ between flaw ② and flaw ③ with inclination angles of 75°, 90° and 105° is simulated, respectively. Figure 14*b* shows the comparison of the crack initiation modes when *β*_2_ is 90°. Wing cracks 1^a^, 1^b^, 1^c^ and 1^d^ are initiated at the left and right ends of the three flaws, and secondary shear cracks 1^e^ and 1^f^ are initiated at the left end of flaw ① and the right end of flaw ②, consistent with the laboratory experimental phenomenon.

Figure 14*c*,*d* shows the propagation process of rock cracks when *β*_2_ values are 75° and 105°, respectively. In figure 13*d*, a transverse crack with a bend in the propagation direction at the left end of flaw ② is observed. The failure mode of the rock changes with the change in the rock bridge inclination. The numerical simulation results are in good agreement with the laboratory tests, indicating the effectiveness of the method used in this study. Notably, there are some differences between the simulated fracture mode and the test results, which may be due to factors such as rock heterogeneity, anisotropy and microcracks in the rock used in the test. In another study [63], we found that the smaller the shape parameter *m* is, the stronger the heterogeneity is, the more serious the damage of the specimen is, and the crack is easier to expand. This conclusion is consistent with the study of Zhang *et al*. [54]. Considering that the main purpose of this paper is to use the improved BB-PD model to study the crack propagation speed, path and coalescence mode of rock specimens containing pre-existing flaws with different number, length, inclination and rock bridge inclination, the differences of simulation results under different shape parameters are not compared, and the relevant research conclusions can be referred to references [54,63].

## 4. Results and discussion

### 4.1. Crack propagation length

Because a material is discretized into elements or particles using a numerical algorithm, previous numerical simulation methods often focused on two aspects: the initiation, propagation and penetration processes of the crack and the stress–strain relationship. However, there are few studies on the propagation distance and fracture length of the crack. The main difficulty in studying this problem is that the actual propagation path of the crack is not a straight line; the crack tip appears forward. Whether in experimental observation or numerical simulation, the propagation length is difficult to record; in particular, the results obtained using conventional continuum mechanics methods, such as the FEM, strongly depend on the size and shape of the grid.

The PD method can not only simulate the mode of crack propagation, but also record the distance of crack propagation in the simulation process [56]. To more specifically compare the effects of different inclination angles on the crack propagation, we studied the changes in the wing crack propagation length under different inclination angles. When simulating the crack propagation process using the PD theory, it is generally stipulated that when the damage value of the material point exceeds a specified value (0.5 in this paper), the distance from this point to the crack initiation point is taken as the crack propagation length [56]. However, in the simulation, the position of the damaged material point at the next time may be closer to the crack initiation point. In this case, the calculated crack propagation length may decrease slightly, but it does not affect the overall variation characteristics of the crack propagation length simulated using the PD method.

**Figure 15.**
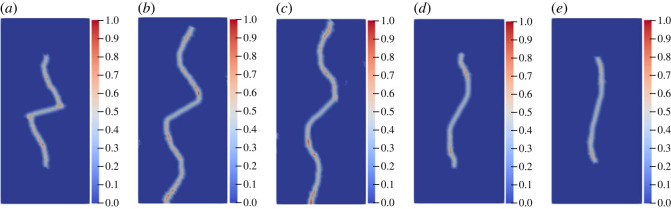
Crack propagation mode with different inclination angles at the step 1580: (*a*) 15°; (*b*) 30°; (*c*) 45°; (*d*) 60°; (*e*) 75°.

The geometric model shown in figure 10*a* is used to conduct uniaxial compression numerical experiments on the specimens with a single flaw under different inclination angles and lengths. When the pre-existing flaw length is constant at 25 mm, the inclination angles are set to 15°, 30°, 45°, 60° and 75°, respectively. The other parameters are the same as those described in §3.2. For the convenience of comparison, only the wing crack propagation is studied. Figure 15 shows the crack propagation mode under different inclination angles when the experiment reaches the step 1580.

**Figure 16.**
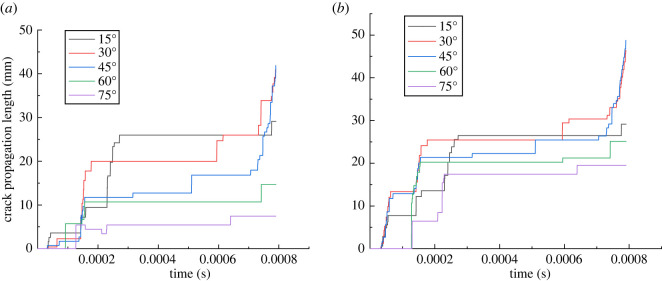
Variation in the wing crack length with time in pre-existing flaw rocks with different inclination angles: (*a*) left; (*b*) right.

Figure 16*a*,*b* shows the change in the wing crack length of the single flaw with different inclination angles. Overall, although the length of the wing crack of the flaw with different angles increases alternately during the propagation process, when the flaw length is constant, whether at the left or right ends of the flaw, the wing crack length increases first and then decreases with the increase in the angle of the flaw after the crack extends to a certain time, consistent with the conclusion reached by Xiao *et al*. [64] through a laboratory experiment. At the step 1580, the crack propagation length is the largest when the inclination angle of the flaw is 45°. Taking the right end of the flaw as an example (figure 16*b*): when the inclination angle increases from 15° to 30° and 45°, the crack propagation length increases from 29.1 mm to 46.5 and 48.7 mm, while when the angle increases to 60° and 75°, the crack propagation length decreases to 25.1 and 19.5 mm in turn.

**Figure 17.**
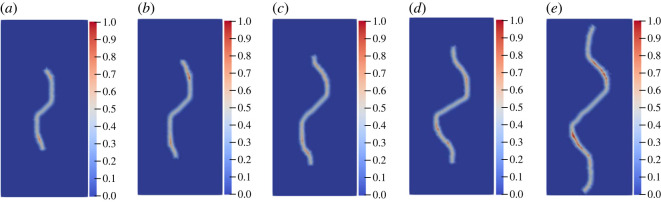
Crack propagation mode with different initial flaw lengths at the step 1300: (*a*) 15 mm; (*b*) 20 mm; (*c*) 25 mm; (*d*) 30 mm; (*e*) 35 mm.

When the inclination angle is constant at 45°, the initial lengths of the pre-existing flaw are set to 15, 20, 25, 30 and 25 mm. Figure 17 shows the changes in the crack propagation mode and propagation length when the simulation reaches the step 1300.

Different from the inclination angle, the length has a significant effect on the crack initiation time: the rock with a longer flaw easily undergoes cracking. After a certain time of crack propagation, the crack propagation length increases with the increase in the pre-existing flaw length. Taking the right end of the first simulated pre-existing flaw as an example, when the length of the pre-existing flaw increases from 15 to 35 mm, the crack propagation time is at step 905, step 70, step 69, step 66 and step 85, and the crack propagation lengths at step 1300 are 19.4, 22.9, 22.3, 30.6 and 38.0 mm, respectively.

**Figure 18.**
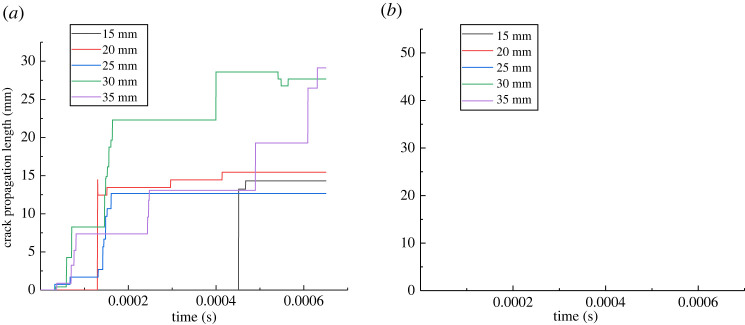
Variation in the wing crack length with time in rocks with a pre-existing flaw with different lengths: (*a*) left; (*b*) right.

From figure 18, when the length of the flaw is small, the crack propagation length may not be different, and there is no evident correlation with the length of the flaw. For example, when the lengths of the pre-existing flaw are 15, 20 and 25 mm in figure 18*b*, the propagation lengths in the step 1300 are 14.3, 15.4 and 12.7 mm, respectively. However, when the lengths of the flaw are 30 and 35 mm, the crack propagation length for almost all the results is significantly higher than that of the other flaw lengths. The reason for this is similar to the above: with the crack gradually expanding to the end, the tensile stress changes into tensile and shear stresses, thereby further increasing the crack propagation length.

### 4.2. Crack coalescence mode of rock with double pre-existing flaws

**Figure 19.**
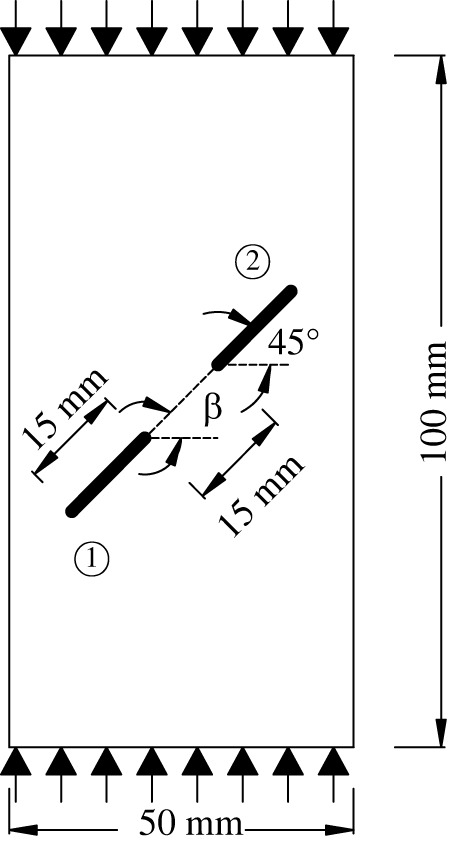
Geometric configuration of a rock with double flaws.

**Figure 20.**
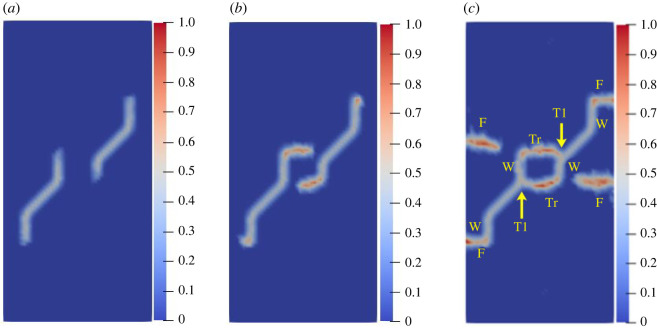
Crack propagation and coalescence process at *β =* 30°: (*a*) step 1500; (*b*) step 1650; (*c*) step 1690.

**Figure 21.**
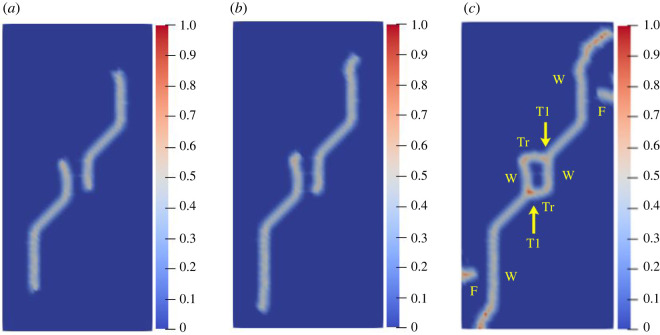
Crack propagation and coalescence process at *β =* 60°: (*a*) step 1500; (*b*) step 1600; (*c*) step 1650.

**Figure 22.**
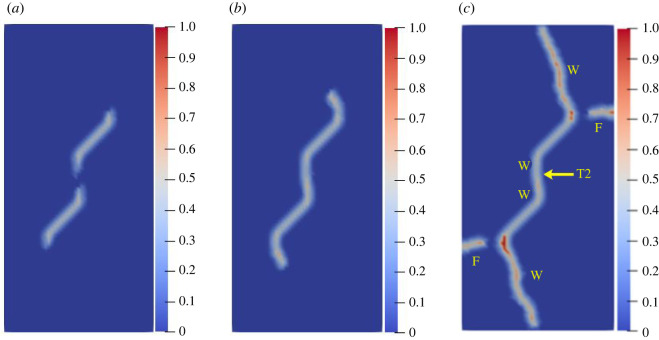
Crack propagation and coalescence process at *β =* 90°: (*a*) step 100; (*b*) step 310; (*c*) step 850.

**Figure 23.**
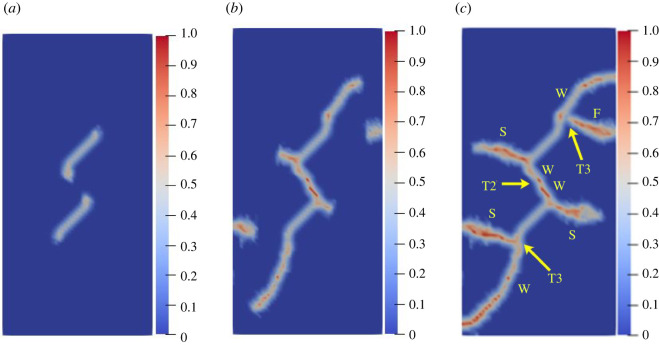
Crack propagation and coalescence process at *β =* 120°: (*a*) step 780; (*b*) step 1920; (*c*) step 1960.

**Figure 24.**
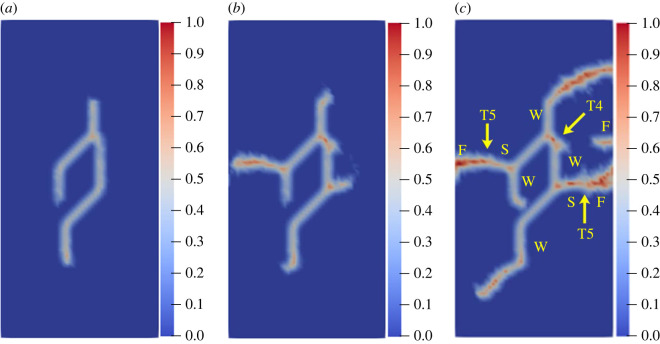
Crack propagation and coalescence process at *β =* 150°: (*a*) step 1500; (*b*) step 1600; (*c*) step 1650.

To study the influence of the rock bridge inclination angle *β* on the crack propagation and coalescence mode, the geometric model shown in figure 19 is used to conduct numerical experiments on rock specimens with double flaws under different rock bridge inclination angles. The length and rock bridge distance of the two pre-existing flaws are constant at 15 mm, and the inclination angle is 45°. The rock bridge inclination angles are set to 30°, 60°, 90°, 120° and 150°, respectively. The other parameters are the same as those described in §3.1. The simulation ends when the crack expands to the upper and lower ends of the rock or the left and right sides of the rock. Figures 20–24 show the results. Due to the size limitation of the figures, letters are used to represent the type of cracks, where ‘W’ represents the wing crack, ‘S' represents the secondary shear crack, ‘Tr’ represents the transverse crack and ‘F’ represents the far-field crack. In addition, T1–T8 is used to represent different crack coalescence forms, and the specific meaning of T1–T8 is explained in table 2.

**Table 2.** Classification of crack coalescence forms.

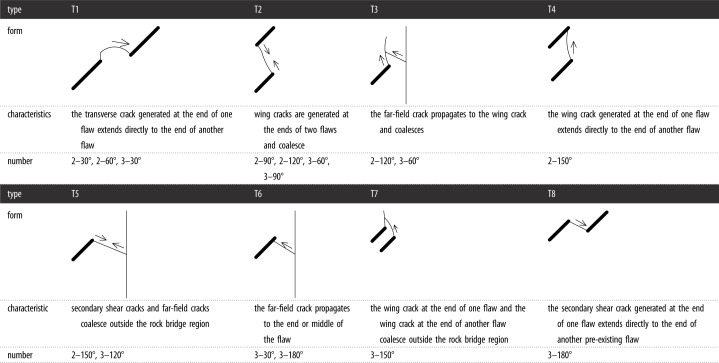

When *β* is 30°, the wing cracks that propagate vertically first appear at both ends of the flaw. The wing cracks at the inner and outer ends of the two flaws propagate gradually. It is not until the step 1650 that wing cracks turn and expand laterally under the action of the shear stress. The crack at the inner end of the flaw propagates laterally faster. At the step 1690, the transverse crack coalesces with the pre-existing flaw, forming an approximately rectangular macrofracture zone. Simultaneously, the wing cracks at the outer ends also extend to the upper and lower ends of the rock, and far-field cracks appear on the left and right sides of the rock.

When *β* is 60°, the wing crack propagation speed increases compared with that at 30°, and the wing crack propagation speed at the outer end of the two flaws is significantly higher than that at the inner end. At the step 1600, the wing crack begins to bend and expand laterally under the action of the shear stress, and the transverse crack coalesces with the inner end of the pre-existing flap at the 1650 time step. Moreover, it forms an approximate rectangular macrofracture zone. At the same time, the wing crack at the outer end of the pre-existing flaw extends to the upper and lower ends of the rock, and small far-field cracks appear on the left and right sides of the rock.

When *β* is 90°, the speed of crack propagation and coalescence is significantly accelerated because of the change in the relative position of the flaws. At the step 310, the wing cracks generated at the inner ends of the two flaws are connected into a straight-line crack. Subsequently, the wing cracks on the outside of the two flaws continue to expand to the upper and lower ends of the rock. Simultaneously, there are far-field cracks on the left and right sides of the rock, and there is a trend of extending to the wing crack.

When *β* is 120°, the wing crack starts to expand relatively gradually. At the step 1920, the wing cracks at the inner end of the flaws link each other, and serious local damage occurs. Simultaneously, secondary shear cracks occur at the inner ends of the two flaws, which has not been observed in the previous three cases. In a very short time (step 1960), the secondary shear crack expands rapidly, and the local damage value is high. The wing cracks at the outer ends of the two flaws also continue to expand rapidly to the left and right sides of the rock, and the far-field cracks on the left and right sides of the rock extend to the wing cracks near the outer end of the flaws, resulting in the second coalescence of the crack.

When *β* is 150°, due to the heterogeneity, the wing crack generated by the lower pre-existing flaw first coalesces with the upper pre-existing flaw. At the step 1600, there are secondary shear cracks at the right end of the two flaws, and there are long and large local damage secondary shear cracks at the left end of the upper flaw. The vertical propagation trend of the two wing cracks also changes. At the step 1650, the wing crack generated by the upper pre-existing flaw extends to the right end of the rock. The secondary shear cracks on the left side of the upper flaw and the right side of the lower flaw connect with the far-field cracks generated at the left and right ends of the specimen. At this time, the wing crack generated on the left side of the upper flaw is close to the lower flaw.

From the above crack propagation process, it can be seen that the wing crack propagation speed at both ends of the pre-existing flaw is relatively low under constant loading, except at the special relative position (rock bridge inclination angle is 90°), and the crack penetration occurs after the step 1500. Once the cracks coalesce for the first time, they propagate faster and extend to the end of the rock in dozens of time steps. In addition, there are wing cracks, transverse shear cracks, and far-field cracks under the different rock bridge inclination angles. However, when the rock bridge inclination angle is greater than 90°, there are shear cracks at the end of the flaw, whereas this phenomenon is not observed when the rock bridge inclination angle is less than 90°. Compared with the wing crack, the secondary shear crack propagates faster, and the local damage value is significantly higher.

### 4.3. Crack coalescence mode of rock with three pre-existing flaws

**Figure 25.**
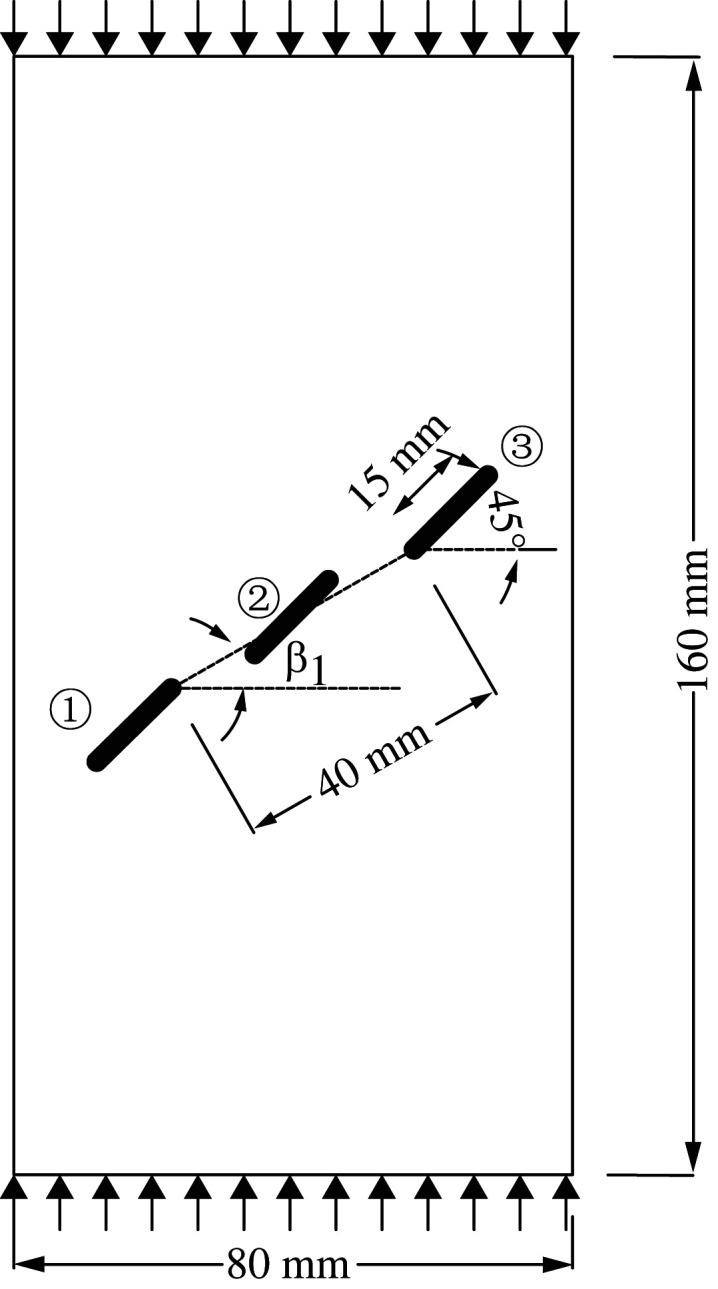
Geometric configuration of rock with three pre-existing flaws.

**Figure 26.**
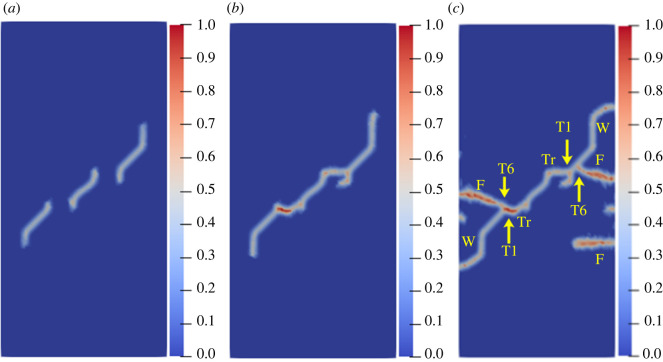
Crack propagation and coalescence process at *β*_1_ *=* 30°: (*a*) step 2650; (*b*) step 2750; (*c*) step 2910.

**Figure 27.**
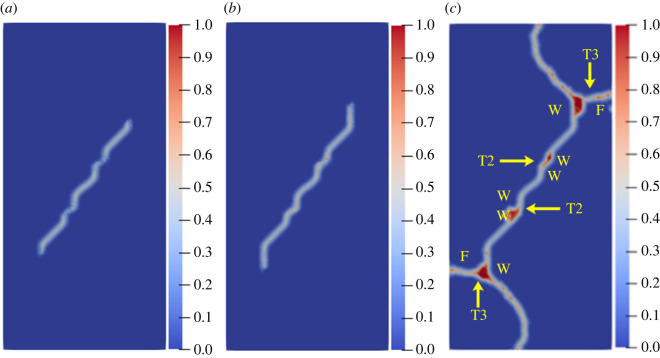
Crack propagation and coalescence process at *β*_1_ *=* 60°: (*a*) step 120; (*b*) step 200; (*c*) step 740.

**Figure 28.**
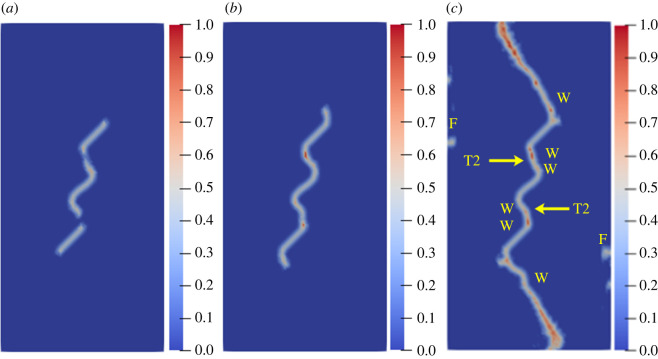
Crack propagation and coalescence process at *β*_1_ *=* 90°: (*a*) step 1000; (*b*) step 1100; (*c*) step 2960.

**Figure 29.**
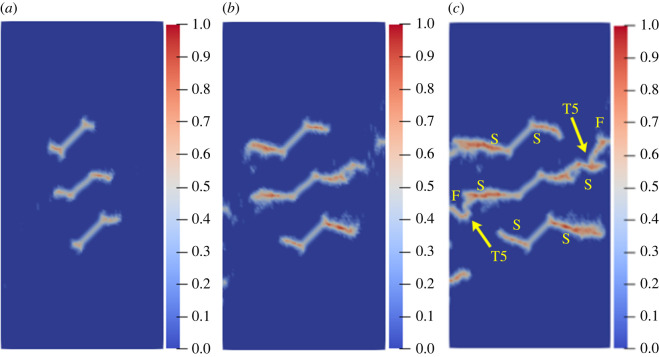
Crack propagation and coalescence process at *β*_1_ *=* 120°: (*a*) step 2800; (*b*) step 2820; (*c*) step 2840.

**Figure 30.**
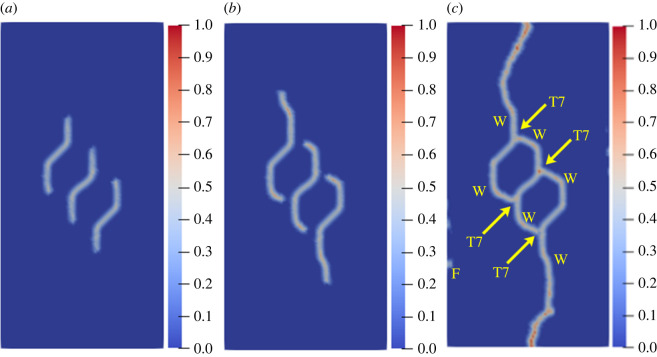
Crack propagation and coalescence process at *β*_1_ *=* 150°: (*a*) step 500; (*b*) step 1650; (*c*) step 2300.

**Figure 31.**
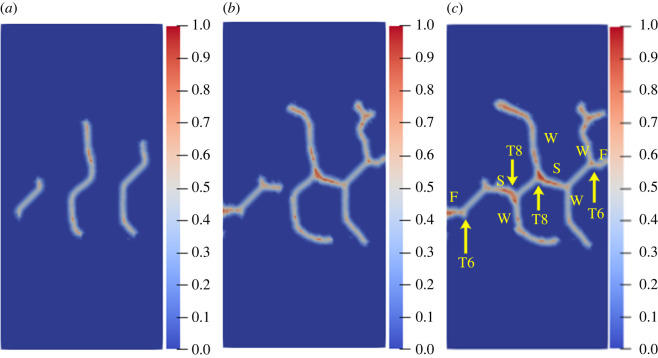
Crack propagation and coalescence process at *β*_1_ *=* 180°: (*a*) step 1500; (*b*) step 2700; (*c*) step 2740.

To further study the influence of different rock bridge inclination angles on the multiple crack propagation and coalescence mode, the geometric model shown in figure 25 is used to conduct uniaxial compression numerical experiments on rock specimens with three flaws with different rock bridge angles. The length of the three pre-existing flaws is 15 mm, and the inclination angle is 45°. The rock bridge spacing for pre-existing flaw ① and pre-existing flaw ③ is fixed at 40 mm, and the rock bridge inclination *β*_1_ is set to 30°, 60°, 90°, 120°, 150° and 180°, respectively. The other parameters are the same as those described in §3.1. The simulation ends when the crack expands to the upper and lower ends of the rock or the left and right sides of the rock; figures 26–31 show the results.

When *β*_1_ is 30°, the propagation speed of each pre-existing flaw is low, and there is no evident wing crack at the crack tip until the step 2650. Subsequently, the crack coalesces for the first time in a short time (step 2750). Specifically, the cracks at the right end of flaw ① and the left end of flaw ② are bent and coalesced, and the crack at the right end of flaw ② propagates laterally and directly to the left end of flaw ③. The wing cracks at the left end of flaw ① and the right end of flaw ③ extend to the left and right sides of the specimen, and the far-field shear cracks on the left and right sides of the rock are directly connected with flaw ① and flaw ③.

When *β*_1_ is 60°, the three pre-existing flaws are approximately on a straight line, the relative position of which is more conducive to the coalescence of wing cracks. At the step 120, there are evident wing cracks at both ends of the flaws. At the step 200, the right end of flaw ① and the left end of flaw ②, and the right end of flaw ② and the left end of flaw ③ are connected. Subsequently, the wing cracks at the left end of flaw ① and the right end of flaw ② continue to expand and extend to the end of the rock at the step 740. At the same time, the far-field cracks on the left and right sides of the specimen connected the wing cracks, and there was serious local damage at the coalesced position.

When *β*_1_ is 90°, the speed of wing cracks at both ends of flaw ② is significantly higher than that of flaw ① and flaw ③, and these coalesce with wing cracks at the upper end of flaw ① and the lower end of flaw ②. The wing cracks at the lower end of flaw ① and the upper end of flaw ③ extend to the upper and lower ends of the rock. At this time, secondary shear cracks are initiated at the lower end of flaw ① and the upper end of flaw ②, and far-field cracks are also initiated on the left and right sides of the rock specimen.

When *β*_1_ is 120°, the left and right ends of the three flaws do not produce wing cracks extending vertically, but produce secondary shear cracks extending laterally just after the wing cracks' initiation. Flaw ② produces secondary shear cracks with the highest growth speed and first coalesces with the far-field shear cracks at the end of the rock. In the entire process, the cracks generated and propagated at the three pre-existing flaws do not coalesce.

When *β*_1_ is 150°, the wing crack propagation speeds at both ends of the three pre-existing flaws are similar, and the wing crack at the left end of flaw ① coalesces with the wing crack at the left end of flaw ② under the combined action of the tensile and shear stresses, and the coalescence position is close to the left end of flaw ②. The wing crack at the right end of flaw ② coalesces with the wing crack at the right end of flaw ①, and the coalescence position is close to the right end of flaw ①. The same crack propagation and coalescence phenomenon also occurred in flaw ② and flaw ③. At the step 2300, an approximately symmetrical hexagonal failure ring is formed between flaw ① and flaw ②, and flaw ② and flaw ③, respectively, and the wing cracks at the right end of flaw ① and the left end of flaw ③ extend to the upper and lower ends of the rock, respectively.

When *β*_1_ is 180°, the wing crack generated at the right end of flaw ② propagates the fastest. Since flaw ① and flaw ③ are close to both sides of the rock, the shear cracks generated by flaw ① and flaw ③ easily extend to the left and right sides of the specimen. At the step 2700, the secondary shear cracks generated at the right end of flaw ② and the left end of flaw ③ coalesce directly. At the step 2740, the secondary shear cracks generated at the right end of flaw ① and the left end of flaw ② coalesce. During the entire process, no wing crack extended to the upper and lower ends of the rock.

The crack coalescence modes in the simulation results of rock with double flaws and triple flaws in this study can be divided into eight categories. Table 2 presents the characteristics and number of each type of the crack coalescence form. For example, 2–30° indicates that it occurs in specimens with double flaws with a rock bridge inclination angle of 30°. In figures 20–24 and 26–31, the crack coalescence form at each position is marked according to the classification in table 1. Rocks with multiple flaws with different rock bridge angles will have different crack coalescence modes, which may be one of the eight forms, for example, two coalescence forms of rock specimens with double flaws with a rock bridge inclination angle of 90° are T2, or it may be a combination of multiple crack coalescence forms. For example, two coalescence forms of rock specimens with three flaws with a rock bridge inclination angle of 30° are a combination of T1 and T3.

From table 2, it can be found that the T2 type is the most common crack coalescence form, which occurs four times in the simulation in this study and often occurs when the flaw is close to the middle of the rock and the wing crack propagation path is approximately a straight line with the rock bridge. T1 type occurs thrice, i.e. when the wing crack initiated at the end of one flaw approaches another pre-existing flaw. T3, T5 and T6 are far-field cracks that coalesce with other different types of cracks. They all appear twice in this paper. T4, T7 and T8 only appeared once. The form of crack coalescence is closely related to the relative position between the flaws and the position of the flaws in rock specimens (near the middle or left and right sides). Additionally, the wing cracks at both ends of the middle flaw are generally the fastest growing in the rock specimen with three flaws, regardless of the rock bridge inclination angle.

## 5. Conclusion

In this study, an improved BB-PD model was developed based on the characteristics of the strain hardening and strain softening of rock under compressive loading. The feasibility of the model was verified by comparing with the results of uniaxial compression failure laboratory tests conducted on an intact rock specimen and rock specimens with single, double and triple pre-existing flaws. Based on the model, numerical experiments were conducted on heterogeneous rocks to study the crack initiation, propagation and coalescence processes of rocks with the flaws for different number, length and inclination angle under uniaxial compression.

(1) In the proposed model, the logarithmic function was used to improve the constitutive model of the bond in the tensile stage, and a quadratic function was used to improve the constitutive model of the bond in the compression stage. Thus, the failure of the bond in the tensile and compression stages could be distinguished on the basis of the characteristics of rock tensile and compression curves.
(2) Wing cracks, secondary shear cracks and far-field cracks often observed in laboratory tests were found in the numerical simulation results, and the crack initiation location, propagation path and coalescence mode were found to be consistent with the previous laboratory tests. The numerical simulation results also showed transverse cracks that were rarely observed in the experiment.
(3) In addition to the later crack initiation at an angle of 75°, the initiation time of the wing crack was unaffected by the inclination angle of the flaw when the flaw length was constant, while when the flaw angle was constant at 45°, the initiation time of the wing crack was significantly earlier with the increase in the flaw length.
(4) When the single flaw length was constant, the length of the wing crack after extending to a certain time increased first and then decreased with the increase in the angle of the flaw. Generally, the crack propagation length was the largest when the angles were 30° and 45°. When the angle was constant, the length of the wing crack after extending to a certain time increased with the increase in the flaw length. This feature was unconspicuous when the pre-existing flaw length was small, but was particularly evident when the flaw length was more than 30 mm.
(5) Eight different crack coalescence modes were found through the simulation of the rock specimens with different angles of the rock bridge. The crack propagation rate after the first coalescence was much higher than that before the coalescence. The crack coalescence mode was influenced not only by the rock bridge inclination angle but also by the relative position of the flaw in the specimen.

## Data Availability

Data in figures 16 and 18 are stored in: https://dx.doi.org/10.5061/dryad.qrfj6q5j5 [65].
